# Healthcare utilisation for febrile diseases in northern Tanzania: a randomised population-based cluster survey

**DOI:** 10.1136/bmjgh-2024-017913

**Published:** 2025-03-06

**Authors:** Deng B Madut, Matthew P Rubach, Julian T Hertz, Rebecca Bodenham, Gwamaka William, Timothy A Peter, Kajiru G Kilonzo, Blandina T Mmbaga, Venance P Maro, John A Crump

**Affiliations:** 1 Department of Medicine, Duke University, Durham, North Carolina, USA; 2 Department of Emergency Medicine, Duke University, Durham, North Carolina, USA; 3 EcoHealth Alliance, New York, New York, USA; 4 Kilimanjaro Christian Medical Center, Moshi, Tanzania; 5 Kilimanjaro Christian Medical College, Moshi, Tanzania; 6 University of Otago, Dunedin, New Zealand

**Keywords:** Health systems, Infections, diseases, disorders, injuries, Community-based survey

## Abstract

**Background:**

We conducted a randomised population-based cluster survey in northern Tanzania to assess care-seeking behaviours in the context of a febrile illness. Our objectives were to determine the most effective points for intervention during initial fever case management and to characterise factors associated with care-seeking.

**Methods:**

The primary sampling unit, or cluster, was the village, and the secondary sampling unit was the household. Villages were selected in a population-weighted fashion, and households were randomly selected within each village. At each household, surveys were administered to determine hypothetical healthcare utilisation patterns for the following febrile syndromes: (1) fever, (2) fever >3 days, (3) fever with diarrhoea, (4) fever with difficulty breathing and (5) fever with confusion. Descriptive analyses were used to evaluate healthcare utilisation for each syndrome at the level of the individual household member, and multivariable logistic regression models were constructed to identify factors associated with care-seeking at a hospital for fever with difficulty breathing or confusion.

**Results:**

From February through October 2018, we enrolled 718 households with a total of 2744 household members. Dispensaries were the leading site of care-seeking for fever (n=1167, 42.5%), fever >3 days (n=1318, 48.0%) and fever with diarrhoea (n=1218, 44.4%). In contrast, hospitals were the leading site for care-seeking for fever with difficulty breathing (n=1436, 52.3%) and fever with confusion (n=1521, 55.4%). Households in the highest wealth quartile had higher odds of reporting that household members would seek hospital care for fever with difficulty breathing or confusion.

**Conclusion:**

In summary, our results suggest that lower-level health facilities, such as dispensaries, play an important role in the initial management of most febrile syndromes. Further research is needed to define the quality of fever case management within such facilities. Finally, our findings provide further evidence that socioeconomic status modifies health-seeking patterns.

**WHAT IS ALREADY KNOWN ON THIS TOPIC**

**WHAT THIS STUDY ADDS**

- We conducted a healthcare utilisation survey and found that lower-level health facilities, such as dispensaries and health centres, play an important role in the initial management of febrile syndromes in northern Tanzania. In addition, we found that households in the highest wealth quartiles were more likely to report that household members would seek hospital care for severe febrile syndromes.

**HOW THIS STUDY MIGHT AFFECT RESEARCH, PRACTICE OR POLICY**

- Our results suggest that further efforts to improve fever case management, including anti-microbial stewardship, should be targeted towards lower-level health facilities. Moreover, our results highlight the importance of collecting socioeconomic data in prospective febrile illness surveillance studies and adjusting disease incidence estimates based on this metric.

## Introduction

 Fever is a common reason for people to seek medical care in sub-Saharan Africa (sSA) and febrile illnesses are a major cause of morbidity and mortality in the region.^1 2^ For this reason, extensive efforts have been made to understand the aetiologies of patients presenting with fever, generally by hospital-based surveillance. While these studies have yielded important insights, many knowledge gaps remain.^1^ One key limitation of hospital-based surveillance is that disease estimates may be biased if individuals who seek care at sentinel surveillance facilities are not representative of the care-seeking behaviours of the population from which they are drawn. To address this limitation, community-based healthcare utilisation surveys (HCUS) have been implemented alongside active surveillance studies.^3^ In addition to adjusting for hospital-based disease estimates, HCUS data may offer crucial insights that inform healthcare planning including where best to intervene to improve initial clinical management of common febrile illnesses.

HCUS are often deployed alongside hospital-based surveillance to adjust disease burden estimates by assessing the proportion of disease unlikely to be captured by surveillance facilities.^3^ By identifying the proportion of participants not captured at sentinel surveillance centres, data collected in HCUS enable population-based disease estimates that adjust for health-seeking patterns. While direct population-based disease estimates can be achieved in large cohort studies, hybrid surveillance strategies offer a lower-cost alternative. HCUS data have also been used to understand barriers to care-seeking. Notably, the available data show that several factors, including age, sex, socioeconomic status (SES) and recognition of disease severity, influence care-seeking behaviour.^4^,^6^ Since delays in care-seeking for some febrile syndromes are associated with poor outcomes,^7^ such data may inform interventions that could improve early recognition of danger signs and timely care-seeking. Furthermore, HCUS data provide an opportunity to document the preferred sites of care-seeking in the context of an illness, allowing for the identification of additional healthcare settings that should be included in disease surveillance. In the context of febrile illnesses, knowledge of preferred sites of care-seeking may also guide other initiatives such as anti-microbial stewardship programmes.

In Tanzania, there is limited understanding of care-seeking behaviours for febrile illnesses, a common reason for seeking healthcare.^8^,^11^ To improve this knowledge, we analysed data from an HCUS conducted in 2018 in which we recruited households in the Kilimanjaro Region of northern Tanzania and evaluated hypothetical care-seeking for febrile illness. We sought to understand preferred sites of care-seeking across different febrile syndromes and the factors that predict seeking hospital care for severe febrile syndromes.

## Methods

### Study setting

**Figure 1:**
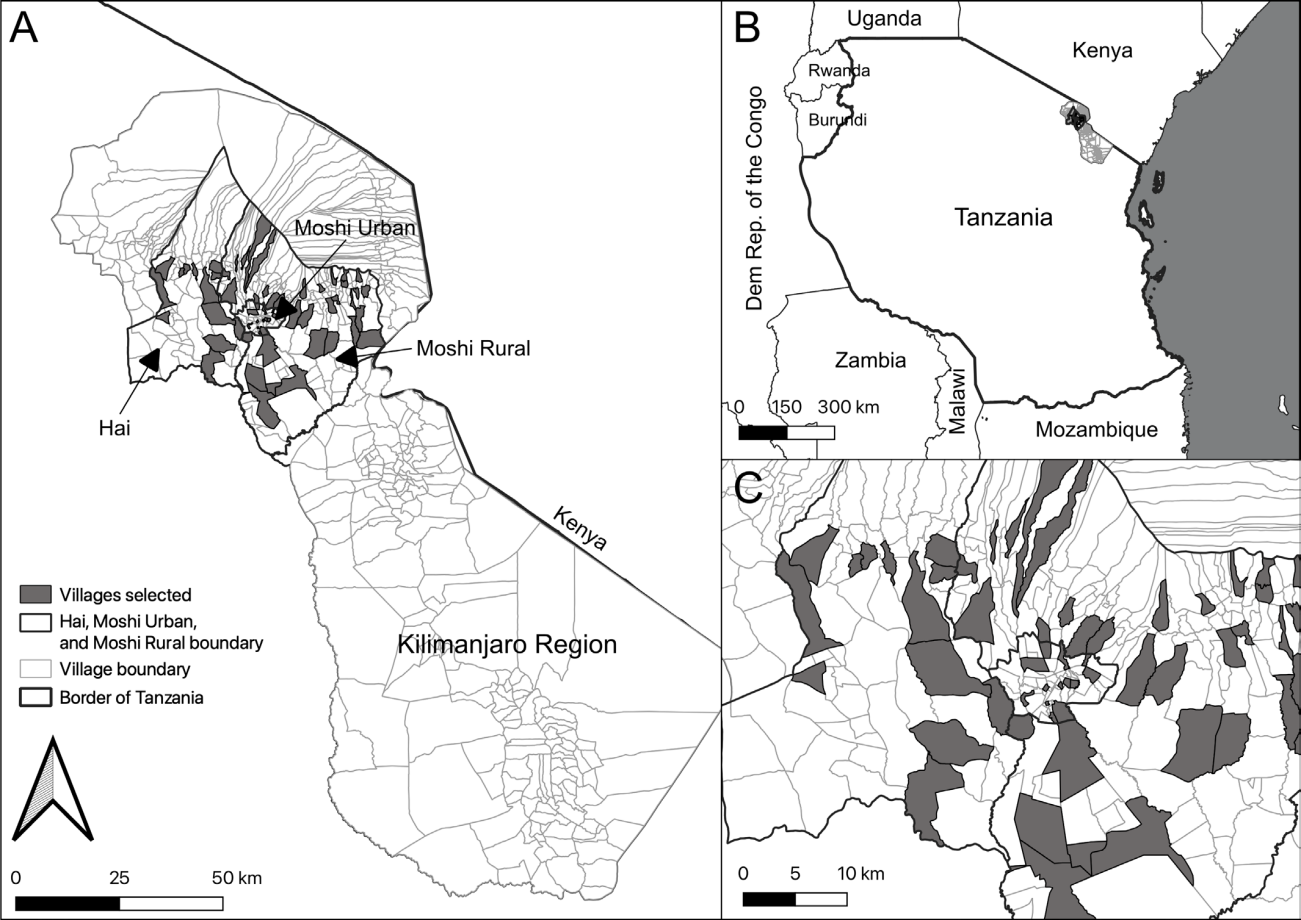
Map of villages enrolled in a randomised population-based cluster survey to evaluate healthcare utilisation for febrile syndromes, Kilimanjaro Region, northern Tanzania, 2018: (A) Kilimanjaro Region and enrolled villages; (B) Tanzania; and (C) districts of Hai District, Moshi Urban District and Moshi Rural District.

The survey was conducted from February 2018 through October 2018 in three districts of the Kilimanjaro Region of northern Tanzania: the urban district of Moshi Urban District (2012 population 184 289^12^) and two surrounding rural districts, Moshi Rural District and Hai District (2012 populations 466 740 and 210 531, respectively^12^) (figure 1). Malaria transmission intensity is low in the Kilimanjaro Region, and HIV seroprevalence among adults aged 15–49 at the time of the survey was 2.2%.^13 14^

### Study design

We conducted a randomised population-based cluster survey based on the WHO guidance for performing vaccination coverage surveys.^15^ The primary sampling unit, or cluster, was the village, and the secondary sampling unit was the household. The survey was conducted in conjunction with a prospective febrile surveillance study at two hospitals in Moshi, Tanzania, from September 2016 through May 2019. The intended objective of the febrile surveillance study was to understand the aetiologies of febrile admissions, including deaths, among patients presenting to two hospitals in northern Tanzania. Some results from the prospective febrile surveillance study have been published.^16^,^21^ The HCUS was conducted to enable adjustments of disease estimates from the prospective febrile surveillance study.^22 23^ The minimum sample size for the HCUS was computed assuming a proportion, defined as the study area’s population that sought healthcare for fever at one of the two study hospitals of 0.1 with a precision of 10%. To be inclusive of households with children under 5 years of age, we conservatively estimated that at least five households would need to be visited to identify one child in this age group. Furthermore, we used an inflation factor of 1.2 to account for non-response, and a design effect of 1.5 was employed to adjust for healthcare utilisation patterns clustering given our study design.^24^ Using these parameters, we calculated that 60 villages with 11 households surveyed from each village would be required.

To select villages, we listed all villages in the districts of Moshi Urban District, Moshi Rural District and Hai District in random order. We then selected villages in a population-weighted fashion based on village-level population data from the 2012 Tanzania Population and Housing Census.^12^ Selected villages are presented in figure 1. Using QGIS 2.18.7, a random starting point was generated in each selected village.^25^ A 1 km^2^ polygon was placed around this starting point, and then 11 random points were generated in this polygon. The *x* and *y* coordinates of these random points were entered into a Garmin eTrex (Garmin Ltd., Olathe, Kansas, USA) hand-held geographical positioning system (GPS). While in the field, the GPS was used to locate the physical location of each random point, and the nearest household to this location was selected for recruitment. If a household refused recruitment, the study personnel proceeded to the next closest household and resumed recruitment per protocol.

### Survey administration

After obtaining informed consent, Tanzanian field workers administered the survey to an adult (age ≥18 y) within the household who self-reported as the household head or a healthcare decision-maker for the household. This individual is hereafter referred to as the ‘respondent’. All survey questions were written in Swahili and English. Responses were entered into Samsung Galaxy Tab A tablets (Samsung, Seoul, South Korea) using Open Data Kit (ODK Inc, San Diego, CA).^26^ Respondents were asked to report the number of household members, age and sex for each household member, health insurance status for individual household members and assets owned by the household. All villages in Moshi Urban District were classified as urban and those in Hai District and Moshi Rural District were classified as rural. This scheme is an administrative classification that aligns with other methods of classifying rural vs urban.^27^

Healthcare utilisation was assessed by asking respondents to determine the first choice of healthcare in the event of the following syndromes for each household member: fever, fever >3 days, fever with diarrhoea, fever with difficulty breathing and fever with confusion. Research assistants underwent extensive training to ensure a consistent understanding of fever syndromes across the study population. First, fever was defined as an elevated body temperature, and research assistants were trained to clearly communicate that the Swahili word for fever, *homa*, referred specifically to its primary meaning—elevated body temperature—rather than its alternative, secondary meaning, which can imply malaise or a general feeling of unwellness. Second, the training focused on standardising the definitions of each febrile syndrome and ensuring clear communication with respondents. Particular emphasis was placed on aligning English descriptions of the syndromes with accurate and culturally appropriate Swahili translations.

**Figure 2:**
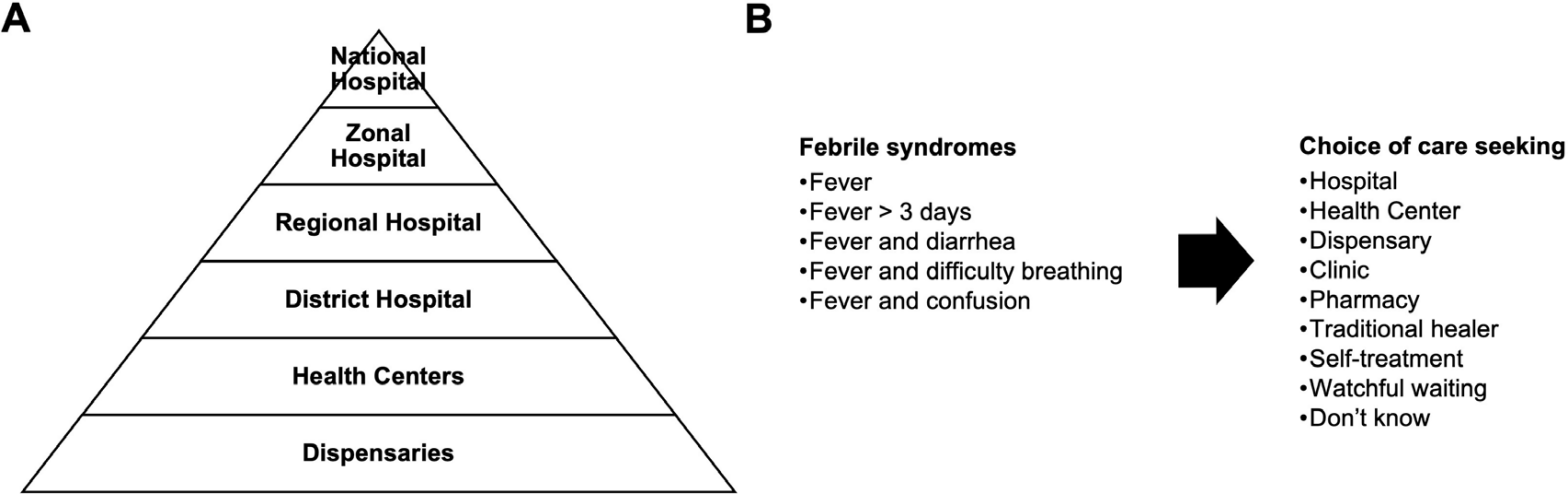
(A) Hierarchy of the health system in Tanzania and (B) choices provided to respondents when asked about behaviours of household members in the setting of five different febrile syndromes, northern Tanzania, 2018.

Predefined categories for assessing healthcare choices were clinic, dispensary, health centre, hospital, pharmacy, self-treatment, traditional healer and watchful waiting (figure 2). Each category was thoroughly explained to respondents to ensure clarity. Self-treatment was defined as managing fever without consulting a health professional (including pharmacists or pharmacy clerks) or traditional healer. Participants were also allowed to respond ‘Don’t know’. Responses of ‘Self-treatment’, ‘Watchful waiting’ and ‘Don’t know’ were recategorised as ‘Did not seek care’ for this analysis.

Categories for care-seeking were intended to reflect the full range of health services available in the public and private sectors of Tanzania.^28^ Briefly, the healthcare system in Tanzania is organised in the form of a pyramid with higher levels of care provided as patients move from bottom to top (figure 2). Persons may seek medical care at any level in this hierarchy based on personal choice. In the public sector, dispensaries represent the lowest level of formal healthcare and provide basic outpatient care. Health centres supervise dispensaries, and while health centres are intended to provide preventative outpatient services, some also provide basic inpatient care. Hospitals provide both outpatient and inpatient services and are categorised as either district, regional, zonal or national, with district hospitals providing the most basic level of hospital-based care.

A private healthcare system exists alongside the public healthcare system and includes private clinics, dispensaries, health centres and hospitals. Pharmacies are generally private entities but are regulated by Tanzanian law, and certain medications, such as most anti-microbials, are intended to be prescription-only. However, studies suggest that a substantial proportion of medications are dispensed without a prescription.^29 30^ Traditional healers also play a role in providing health services, particularly in communities where access to conventional health services is not readily available.^31 32^ Health services provided by traditional healers often differ in their concept of the cause of disease and their approach to treatment when compared with evidence-based medicine.^31^

### Patient and public involvement

Patients and members of the public were not directly involved in the design or conduct of this study.

### Statistical analyses

Statistical analyses were performed in R Statistical Software (v4.3.2; R Core Team 2023) and Stata V.18.0 (StataCorp, College Station, TX). Continuous variables were expressed using medians and IQR, and categorical variables were expressed as frequencies. Care-seeking for each syndrome was evaluated at the level of the individual household member. A SES score was derived via principal component analysis (PCA), following the methodology used in national Demographic and Health Surveys (DHS) to generate a wealth index.^33^ Our approach involved household ownership of the following assets: ownership of radio, ownership of television, ownership of a motorcycle, ownership of a car, piped water to home, presence of a flush toilet in the home, presence of electricity in the home, ownership of a refrigerator, ownership of a bank account, type of wall material (brick, cement or concrete, or tiles) in the home and type of floor material (brick, cement or concrete, or tiles) in the home. The PCA-generated SES score was then divided into quartiles to stratify the study population into four groups, representing increasing levels of wealth. Multivariable logistic regression was used to determine predictors for seeking care at a hospital for fever with difficulty breathing or confusion since guidelines characterise these syndromes as ‘severe’ and recommend prompt care-seeking at a hospital.^34 35^ Hospital care-seeking for fever with difficulty breathing or confusion was evaluated at the household level with households categorised as seeking care at a hospital if respondents reported that at least one household member would seek care at a hospital for that syndrome. Independent variables included in each multivariable model were selected a priori.

## Results

### Household and respondent characteristics

We enrolled 718 households, of which 154 (21.4%) were in an urban setting and the median (IQR) household size was four (two to five) persons. All households included at least one household member >15 years old, 315 (43.9%) reported at least one household member 5 to 15 years old and 230 (32.0%) reported at least one household member <5 years old. Of respondents, median (IQR) age was 48 (31–63) years, 500 (69.6%) were female and 157 (21.9%) reported their education level as secondary or above. Household and respondent characteristics are presented in table 1.

**Table 1:** Characteristics of 718 households in a randomised population-based survey to evaluate healthcare utilisation for febrile syndromes, northern Tanzania, 2018

Variables	n	(%)*
Household size, median (IQR)	4	(2–5)
Respondent age, median (IQR)	48	(31–63)
Respondent age		
< 40 years	270	(37.6)
≥ 40 to < 60 years	230	(32.0)
≥ 60 years	218	(30.4)
Respondent gender		
Male	218	(30.4)
Female	500	(69.6)
Age groups within households		
< 5 years	230	(32.0)
5 to 15 years	315	(43.9)
> 15 years	718	(100)
Respondent education		
Primary or less	561	(78.1)
Secondary or above	157	(21.9)
Residence		
Rural	564	(78.5)
Urban	154	(21.5)
Has health insurance†	224	(31.2)
Household ownership		
Phone	657	(91.5)
Type of floor material‡	591	(82.3)
Type of wall material§	574	(79.9)
Radio	527	(73.4)
Tap water	473	(65.9)
Electricity	427	(59.5)
Flush toilet	301	(41.9)
Television	297	(41.3)
Iron	238	(33.2)
Bank account	142	(19.8)
Refrigerator	128	(17.8)
Motorcycle	71	(9.9)
Car	44	(6.1)

* Data presented as n (%) unless otherwise specified.

† At least one household member has health insurance.

‡ House floor made of brick, cement/concrete, or tiles.

§ House wall made of brick, cement/concrete, or tiles.

### Hypothetical care-seeking behaviour for febrile syndromes for household members

**Figure 3:**
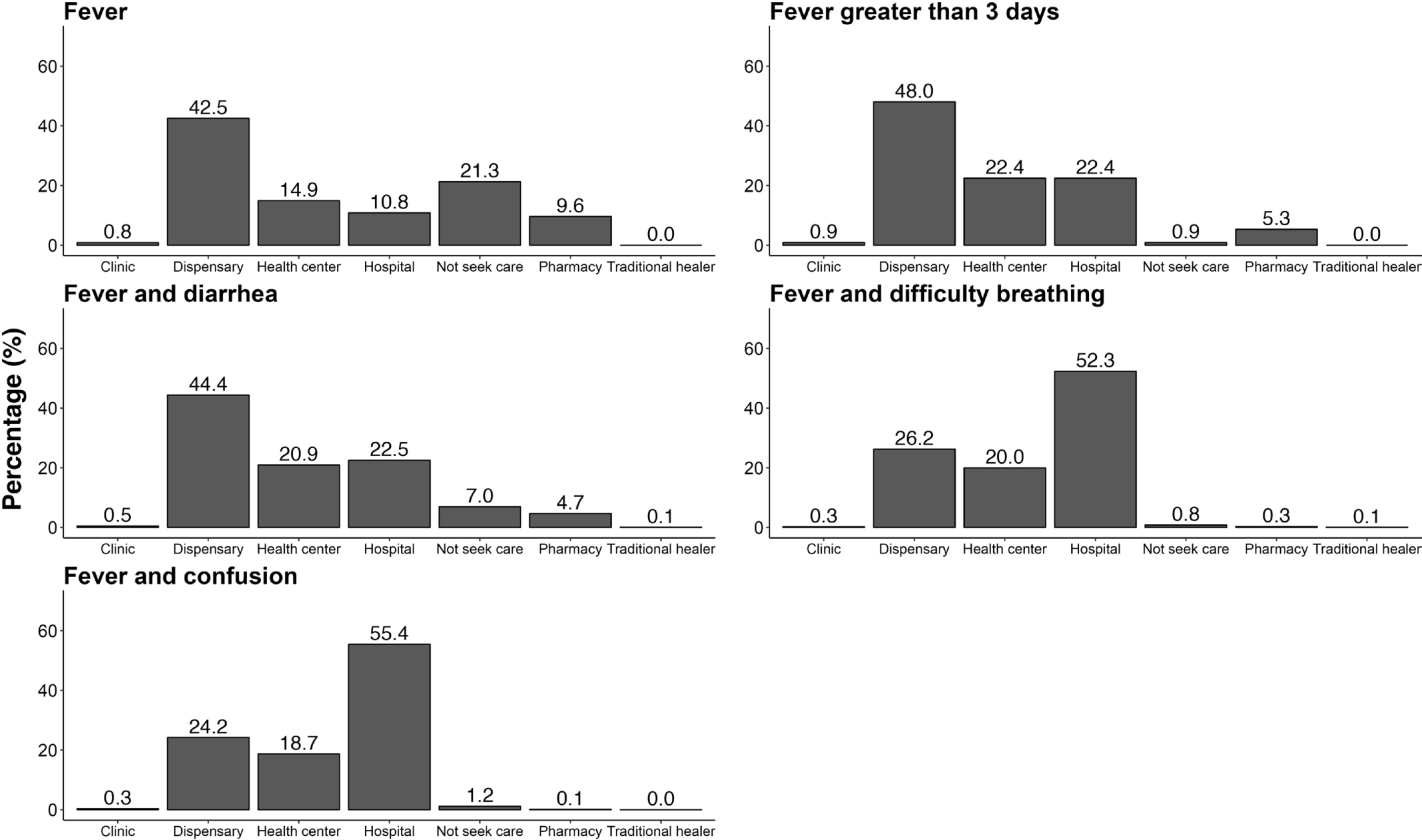
Site of care-seeking for hypothetical febrile syndromes among 2744 household members enrolled in a multistage randomised population-based survey to evaluate healthcare utilisation for febrile syndromes, Kilimanjaro Region, northern Tanzania, 2018.

Care-seeking behaviour was analysed at the level of the household member. Among the 718 households, there were 2744 household members. Dispensaries were the leading site of care-seeking for fever (n=1167, 42.5%), fever >3 days (n=1318, 48.0%) and fever with diarrhoea (n=1218, 44.4%). In contrast, hospitals were the leading site for care-seeking for fever with difficulty breathing (n=1436, 52.3%) and fever with confusion (n=1521, 55.4%). Traditional healers were the least frequent site of care-seeking across all febrile syndromes and were only reported as a site of care-seeking for fever with diarrhoea (n=2, 0.1%), fever with difficulty breathing (n=2, 0.1%) and fever with confusion (n=1, <0.1%). The sites of hypothetical care-seeking are presented in figure 3 and online supplemental table 1.

### Predictor of seeking hospital care

Predictors of care-seeking at a hospital for fever with difficulty breathing or confusion are presented in table 2. Respondent aged over 60 years was associated with lower odds of reporting that household members would seek hospital care for fever with difficulty breathing (adjusted OR (aOR), 0.47, 95% CI 0.32 to 0.69) and fever with confusion (aOR 0.49, 95% CI 0.33 to 0.72). Female gender of respondents was associated with lower odds of reporting that household members would seek hospital care for fever with difficulty breathing (aOR 0.54, 95% CI 0.38 to 0.77) and fever with confusion (aOR 0.58, 95% CI 0.41 to 0.82). Compared with rural households, urban households were associated with lower odds of hospital care-seeking for fever with difficulty breathing (aOR 0.61, 95% CI 0.41 to 0.92). Households in the highest wealth quartile were associated with hospital care-seeking for fever with difficulty breathing (aOR 2.16, 95% CI 1.35 to 3.44) and fever with confusion (aOR 2.18, 95% CI 1.37 to 3.49).

**Table 2:** Predictor of seeking care at a hospital for febrile syndromes among 718 households enrolled in a randomised population-based survey to evaluate healthcare utilisation for febrile syndromes, northern Tanzania, 2018

Variables	Model 1aOR (95% CI)	Model 2aOR (95% CI)
Respondent age		
< 40 years	Reference	Reference
≥ 40 to < 60 years	0.82 (0.56 to 1.18)	0.77 (0.53 to 1.11)
≥ 60 years	0.47 (0.32 to 0.69)	0.49 (0.33 to 0.72)
Respondent gender		
Male	Reference	Reference
Female	0.54 (0.38 to 0.77)	0.58 (0.41 to 0.82)
Respondent education		
Primary or less	Reference	Reference
Secondary or more	1.30 (0.87 to 1.94)	1.11 (0.74 to 1.65)
Residence		
Rural	Reference	Reference
Urban	0.61 (0.41 to 0.92)	0.70 (0.46 to 1.05)
Wealth quartiles		
First	Reference	Reference
Second	1.10 (0.72 to 1.68)	0.92 (0.60 to 1.40)
Third	1.34 (0.88 to 2.05)	1.30 (0.85 to 1.98)
Fourth	2.16 (1.35 to 3.44)	2.18 (1.37 to 3.49)

Model 1: Ffever with difficulty breathing.

Model 2: Ffever with confusion.

aOR, adjusted OR

## Discussion

We characterised healthcare utilisation behaviours across multiple febrile syndromes for households in northern Tanzania. We observed that dispensaries and health centres were the leading sites of care-seeking for fever, fever >3 days and fever with diarrhoea. In contrast, hospitals were the leading site for care-seeking for fever with difficulty breathing and fever with confusion. Predictors of care-seeking at a hospital differed across syndromes and included the age and gender of the respondent, rural versus urban residence and SES.

We observed that dispensaries and health centres were leading sites for seeking care across all febrile syndromes—a pattern consistent with prior research in Tanzania.^11^ However, knowledge of the quality of fever case management within these facilities is not well characterised. Existing data point to several challenges, including inadequate drug supply, delays in referring patients with severe illness to hospital-level care, understaffing, and presumptive anti-malarial and anti-bacterial prescribing without diagnostic testing.^36^ Given the central role of these facilities in the initial management of fever, studies are needed to assess the quality of care they provide, particularly regarding its impact on health outcomes and implications for anti-microbial stewardship efforts. In addition, our findings, along with previous research, suggest that dispensaries and health centres could serve as valuable sites for infectious disease surveillance studies.^11^ Their inclusion is likely to provide a more comprehensive understanding of the spectrum of aetiologies contributing to febrile illness in our setting.

A higher percentage of respondents reported that household members would seek hospital care for fever with difficulty breathing or confusion compared with other syndromes. It is possible that respondents appropriately perceived these syndromes as more severe and therefore requiring a higher level of care. However, it should be noted that data on patient risk perception of febrile syndromes in our setting and broadly in sSA are limited.^37 38^ In our multivariable regression models, we found that age >60 years and female gender of the respondent were associated with lower odds of reporting that household members would seek hospital care for fever with difficulty breathing or confusion. Prior studies report mixed findings on the relationship between age, gender and healthcare decision-making and care-seeking behaviour. For instance, younger healthcare decision-makers may be less adept at recognising danger signs than older decision-makers, potentially leading to delayed care-seeking.^39^ Conversely, anecdotal evidence suggests that older adults in sSA delay seeking care across various illnesses for a range of reasons, including social isolation.^40 41^ Regarding gender, women tend to more often appropriately seek care for themselves, and on behalf of others as healthcare decision-makers, than men.^42^ However, some studies show that delays in care-seeking may occur for some female healthcare decision-makers because of the tendency to involve other family members in decision-making.^39 43 44^

We also found that the odds of seeking care at a hospital were higher for respondents from households in the highest wealth quartile for fever with difficulty breathing or confusion. This finding is not unexpected since wealthier households in sSA are consistently shown to display higher care-seeking behaviour at health facilities, particularly hospitals.^4 45^ Our findings underscore the importance of collecting socioeconomic data in hybrid surveillance studies designed to generate population-based incidence estimates for infectious diseases.^3^

Some findings were unexpected and warrant further discussion. First, traditional healers were not reported as a major site of care-seeking across febrile syndromes. Prior research has shown a higher prevalence of use of traditional healers in Tanzania.^31^ Reasons that our results differ may include social desirability bias and a mixed approach to care-seeking wherein traditional healers are sought only when illness persists despite conventional health services.^46^ Second, we observed that the odds of seeking care at a hospital for fever with difficulty breathing were lower for respondents from urban households. It is important to recognise that residents of urban areas in Tanzania show disadvantages across many health outcomes compared with those in rural areas.^47 48^ Poor health outcomes for urban residents in Tanzania may partly be explained by the unintended consequences of rapid urbanisation such as poorer air quality and limited access to clean water. Despite this, the general perception is that urban residents have greater access to formal medical care, including hospital care. Therefore, further research is needed to verify our findings and, if confirmed, elucidate the causal pathways underlying this association.

Our study had several limitations. First, our analysis was based on healthcare-seeking behaviour preferences ascertained through hypothetical clinical scenarios. Prior research shows that survey respondents may overestimate their intended healthcare utilisation when provided with hypothetical scenarios.^49^ However, assessing actual care-seeking may be equally constrained by the risk of recall bias.^50^ Second, it is important to note that HIV prevalence and malaria transmission were low in our setting compared with some other locations in Tanzania or sSA. As these factors may influence care-seeking behaviour,^51^,^53^ our findings may not be generalisable to other settings with a higher HIV prevalence or malaria transmission. Third, our regression models evaluating predictors of hospital care-seeking did not include variables previously shown to influence care-seeking behaviour. These include travel distance to the nearest hospital, perceived quality of health providers, and disease knowledge.^54^,^56^

In summary, our data suggest that a substantial proportion of febrile patients in our setting present to facilities outside of hospitals, particularly dispensaries and health centres. This finding suggests that efforts to assess and improve fever case management and community anti-microbial stewardship may be targeted to these settings. Moreover, leveraging these facilities as surveillance sites in future prospective studies may provide a more comprehensive knowledge of febrile illness epidemiology. Finally, our findings provide further evidence that adjusting for SES may improve the accuracy of disease incidence estimates.

## supplementary material

10.1136/bmjgh-2024-017913 online supplemental table 1

## Data Availability

Data are available upon reasonable request.
