# Supplementary material for: Healthcare utilisation for febrile diseases in northern Tanzania: a randomised population-based cluster survey
Source: BMJ Glob Health. 2025 Mar 6;10(3):e017913. doi: 10.1136/bmjgh-2024-017913 (PMC11887305; doi:10.1136/bmjgh-2024-017913)
Supplement: online supplemental table 1 [file bmjgh-10-3-s001.pdf]

Supplementary Table 1. Site of care-seeking for hypothetical febrile syndromes among 2,744 household members enrolled in a multistage randomized population-based survey to evaluate healthcare utilization for febrile syndromes, Kilimanjaro Region, northern Tanzania, 2018

Facilities	Fever		Fever > 3 days		Fever with diarrhea		Fever and difficulty breathing		Fever and confusion	
	n	(%)	n	(%)	n	(%)	n	(%)	n	(%)
Hospital	297	(10.8)	616	(22.4)	618	(22.5)	1436	(52.3)	1521	(55.4)
Health center	409	(14.9)	616	(22.4)	574	(20.9)	548	(20.0)	513	(18.7)
Dispensary	1167	(42.5)	1318	(48.0)	1218	(44.4)	720	(26.2)	664	(24.2)
Clinic	23	(0.8)	24	(0.9)	13	(0.5)	7	(0.3)	9	(0.3)
Pharmacy	264	(9.6)	146	(5.3)	128	(4.7)	9	(0.3)	4	(0.1)
Not seek care	584	(21.3)	24	(0.9)	191	(7.0)	22	(0.8)	32	(1.2)
Traditional healer	0	(0)	0	(0.0)	2	(0.1)	2	(0.1)	1	(0.0)
